# Simple Risk Score for Prediction of Early Recurrence of Hepatocellular Carcinoma within the Milan Criteria after Orthotopic Liver Transplantation

**DOI:** 10.1038/srep44036

**Published:** 2017-03-09

**Authors:** Jiliang Feng, Jushan Wu, Ruidong Zhu, Dezhao Feng, Lu Yu, Yan Zhang, Dayu Bu, Chenlei Li, Yuyan Zhou, Lianghao Si, Yuhan Liu, Ziwei Liang, Jianing Xu, Tianjun Wu

**Affiliations:** 1 Clinical-Pathology Center, Bejing You-An Hospital, Capital Medical University, Beijing, China; 2 General Surgical Center, Bejing You-An Hospital, Capital Medical University, Beijing, China; 3 College of Life Science, Sichuan University, Sichuan, China; 4 Medical Record Statistics Management Center, Bejing You-An Hospital, Capital Medical University, Beijing, China.

## Abstract

Ten to twenty percent of the hepatocellular carcinoma (HCC) patients fulfilling the Milan criteria (MC) recurred within three years after orthotopic liver transplantation (OLT). We therefore utilize a training cohort to develop an improved prognostic model for predicting the recurrence in these patients. By univariate and multivariate analysis, AFP level [cut-off value: 321 ng/mL, area under the curve (AUC) = 0.724, 95% confidence interval (CI) = 0.604–0.843, P < 0.001] and cytokeratin-19 (CK19) and glypican-3 (GPC3) expression pattern from nine putative prognostic factors were entered in risk factor scoring model to conjecture the tumor recurrence. In the training cohort, the AUC value of the model was 0.767 (95% CI = 0.645–0.890, P < 0.001), which was the highest among all the elements. The model’s performance was then assessed using a validation cohort. In the validation cohort, the AUC value of the model was 0.843 (95% CI = 0.720−0.966, P < 0.001) which was higher than any other elements. The results indicated that model had high performance with good discrimination ability and significantly improved the predictive capacity for the recurrence of HCC patients within MC after OLT.

Currently, orthotopic liver transplantation (OLT) is recognized as an excellent treatment approach for hepatocellular carcinoma (HCC) in well-selected candidates, and the Milan criteria (MC) is commonly applied as a basis for the patient selection before transplantation, worldwide. Although some investigators have argued that MC was too restrictive and limited the transplantation option, approximately 10–20% of the patients within this standard recurred after transplantation^1^,^2^,^3^. Thus, identifying individuals with high recurrence risk could aid in not only the evaluation of prognosis but also in developing selection criteria and optimizing treatment strategies.

The establishment of MC was based on the tumor macro-morphology, including only the tumor size and number. Therefore, HCC patients with aggressive biological behavior could have developed extrahepatic dissemination before LT even fulfilling the MC. Today, it is widely recognized that biomarkers that are closely linked to the aggressive biology of tumor cells possess a great potential as diagnostic and prognostic tool in molecular medicine.

The adult liver is composed of dominant mature hepatocytes. Thus, for an extended period, the primary carcinomas of the liver have been speculated to have originated from the mature hepatic parenchymal cells (by dedifferentiation). With the understanding of the hierarchical constitution of the parenchymal cells in the adult liver, it has now been realized that HCC comprised of a heterogeneous group of subtypes, which may have transformed from hepatic progenitor cell (HPC), as well as the HPC-derived progenies towards maturation^4^,^5^. Cytokeratin-19 (CK19) and glypican-3 (GPC3) are both routine pathological diagnosis biomarkers for the primary carcinoma of the liver. CK19 has been used as a differentiation diagnostic marker for cholangiocellular carcinoma from HCC. However, the expression of the protein in some HCC subtypes^6^,^7^ and hepatoblastoma^8^ indicated that the CK19 positive HCC could originate from the HPC. Similar to alpha-fetoprotein (AFP), GPC3 is also a fetoprotein. It is abundantly expressed in HPC^9^,^10^, as well as immature hepatocytes^11^. The expression of GPC3 is absent during the terminal differentiation period of HPC close to mature hepatocyte^9^. This phenomenon varies from the expression spectrum of CK19, because when HPC is committed to the hepatocyte lineage, the expression of CK19 is decreased at the very early stage^12^,^13^,^14^. Therefore, according to the particular expression phase and spectrum of CK19 and GPC3 in the differentiation process of HPC towards mature hepatocytes, the phenotypes of CK19+/GPC3+, CK19−/GPC3+, and CK19−/GPC3− can approximately corresponded to the HPC/hepatoblast, immature hepatocyte, and terminally differentiated hepatocyte, respectively. Consecutively, the hierarchal HCC subtypes that may have been transformed from the various differentiation stages of hepatic parenchymal cells can fall into the CK19+/GPC3+, CK19−/GPC3+, and CK19−/GPC3− immune-phenotype groups, respectively.

The correlation between the HCC subtyping based on CK19 and GPC3 combined detection and tumor biology had been identified earlier^15^. It was observed that patients with CK19+/GPC3+ expression carried the highest risk of microvascular invasion, regional lymph node involvement, and intrahepatic and distant metastasis, followed by patients with CK19−/GPC3+, and finally the CK19−/GPC3− phenotype. The finding implied that the cellular differentiation status was closely linked to the aggressive biology of tumor cell in HCC. Although the prognostic significance of GPC3 or CK19 in patients with HCC has been emphasized, in view of the previous findings, we speculated that by combining the detection of CK19 and GPC3, a rather comprehensive sub-classification of HCC can be defined, which might develop an efficient stratification of the prognosis of HCC patients. Since the majority of the recurrence of the tumor occurs within the first two years after LT, the present study was designed to observe the correlation between the sub-classification of HCC based on the CK19 and GPC3 combined detection and early recurrence of patients who conformed to MC and underwent OLT. This would provide an insight into the development of an improved prognostic model for predicting recurrence in these patients.

## Subjects and Methods

### Subjects

Organ donation or transplantation in the study was strictly implemented under the regulation of the China Organ Donation Committee (CODC), Organ Transplant Committee (OTC), and the Declaration of Helsinki (1983). Informed consent was obtained from the subjects. The study protocol was approved by the Ethics Committee of Beijing You-An Hospital, Capital Medical University.

**Figure 1:**
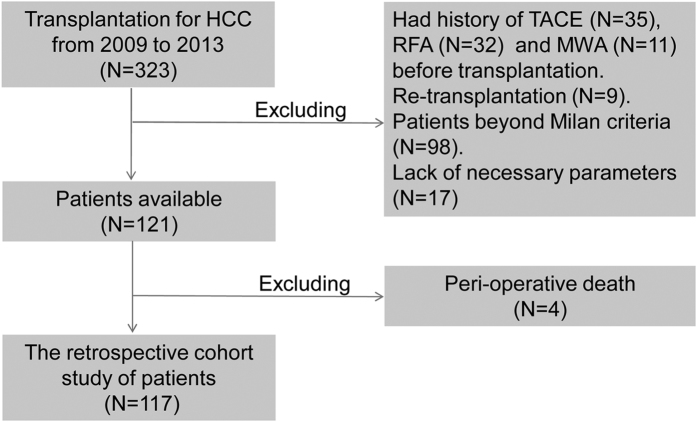
Flow chart of patient selection procedures in training cohort. HCC, hepatocellular carcinoma; TACE, transarterial chemoembolization; RFA, radiofrequency ablation; MWA, microwave ablation.

Between January 2009 and October 2013, a total of 323 HCC patients received OLT at You-An Hospital, Capital Medical University, and their records were reviewed. According to the selection criteria (Fig. 1), 206 HCC patients were excluded, and the remaining 117 HCC patients formed a training data set to develop an improved prognostic model for predicting the recurrence in these patients. Based on the same selection criteria, another independent validation cohort of 51 patients from January 2005 to December 2008 was used to assess the performance of the predicting model.

### Surgical procedures

All surgeries were performed by the same groups of doctors. Patients in accordance with the MC and without other severe and serious organ diseases were eligible for participation in the study. MC was defined as one tumor ≤5 cm, or two or three tumors with each tumor ≤3 cm, without any vascular invasion or metastasis as observed by computed tomography^16^. OLT was performed with an ABO-compatible liver graft, using the same technique in all the patients. Triple regimen immunosuppression was followed, which consisted of tacrolimus capsules (FK506, Astellas Ireland Co. Ltd., Dublin, Ireland), mycophenolate mofetil (MMF, Shanghai Roche Pharmaceuticals Ltd., Shanghai, China), and methylprednisone (Pfizer Manufacturing Belgium NV, Puurs, Belgium)^17^. Methylprednisolone (10 mg/kg) was intravenously infused during operation. After surgery, it was given by 200 mg/day, and daily reduced by 20% till the 6^th^ day. Then it was changed to oral (16 mg/day), and weekly reduced by 25% till the 4^th^ week (4 mg/day), and keep this dose for 2 months till withdrawn. FK506 was added at the 24^th^ hours after operation, and the initial dose was 0.1 mg/(kg.d), then maintained the drug concentration at 8–10 ng/mL within 3 months after surgery. Subsequent adjustments in FK506 dosage were made depending on the hepatic graft function, adverse effects from the drug, and the drug’s plasma trough levels. MMF was given by 1000 mg/day after operation, and withdrawn 3 months later. Any other anticancer treatment was not administered to the patients until recurrence.

### AFP measurement

The serum AFP concentrations were analyzed using the Elecsys 2010 System (Roche Diagnostics, Mannheim, Germany) according to the manufacturer’s instructions. The pre-transplant serum was collected and analyzed (measured within 24 h preceding transplantation). In this work, the last available pre-transplant serum was studied as a prognostic indicator.

### Immunohistochemical staining

Hematoxylin and eosin-stained slides and formalin-fixed-paraffin-embedded blocks of HCC from OLT patients were retrieved from the archives of the Department of Pathology, Beijing You-An Hospital, Capital Medical University. The immunohistochemistry (IHC) was performed as described previously^18^. Mouse anti-human CK19 monoclonal antibody (Clone BA17; Dilution, 1:100) and mouse anti-human GPC3 monoclonal antibody (Clone 1G12; Dilution, 1:200) was purchased from Zeta Company (Sierra Madre, CA, USA). The samples were subjected to the steamer for 20 min in citrate target retrieval buffer (pH 6.0). The results of IHC were interpreted as positive if greater than 5% of the tumor cells showed cytoplasmic staining for CK19 or GPC3. All the enrolled cases were divided into three groups: CK19+/GPC3+ group, wherein tumor cells co-expressed CK19 and GPC3; CK19−/GPC3+ group including cases wherein tumor cells solely expressed GPC3; CK19−/GPC3− group, without the expression of both CK19 and GPC3. The evidence of cytoplasmic staining of adjacent interlobular duct epithelia served as an internal positive control for CK19, whereas the yolk sac tumor tissue was used as a positive control sample for GPC3. Negative controls were carried out by substitution of the primary antibodies with a non-immunized serum that resulted in no signal detection. The expression of these markers was assessed independently and blindly by two investigators. All the slides were reviewed to confirm the diagnosis according to the guidelines of World Health Organization (WHO) criteria, 2010^19^.

### Follow-up

All patients were followed-up at our outpatient clinic or through a telephonic interview. The surviving patients were regularly followed-up at the clinic: monthly during the first six months after OLT, every three months from the 7^th^ to the 18^th^ month, and every six months after that. Abdominal and pelvic ultrasonography and chest X-ray were performed as routine examinations. When metastasis or recurrence was suspected, further evaluations were made by contrast-enhanced computed tomography or magnetic resonance imaging scan and, if necessary, by ultrasound-guided biopsy to confirm the diagnosis. The endpoint of the present study was the estimation of recurrence-free survival (RFS) time. The RFS time was calculated from the date of OLT to the date of a suspected tumor recurrence in patients with eventually confirmed tumor recurrence or up to the last follow-up contact in patients without tumor recurrence. Patients who died before experiencing the disease recurrence or were loss of follow up were considered censored. The cut-off date of the follow-up was August 1, 2016.

### Statistical analysis

All analyses were performed using the SPSS (version 21.0, SPSS, Chicago, IL, USA). The categorical data were described by frequency and percentage, whereas continuous data by mean ± SD or median (range). The χ^2^ test and Student’s t-test was applied to compare the distribution of categorical and continuous variables, respectively. The survival curves for the patients were plotted using the Kaplan-Meier method and differences between the curves were assessed using the log-rank test. With the utilization of the ROC curve, the cut-off value of serum AFP level for recurrence and the area under the ROC curve (AUC) was determined, and the Youden’s index calculated. Univariable and multivariable Cox proportional hazards regression analysis was used to assess the factors associated with recurrence. Before performing multivariate analyses, the significant factors in the univariate analysis were assessed for multicollinearity. A tolerance <0.20 and/or a variance inflation factor (VIF) of ≥10 indicates a multicollinearity problem^20^,^21^. A scoring model was devised by multivariable Cox regression analytical results from the training cohort. We assigned a risk score for each variable based on its coefficient value, standardized with the lowest value, which was assigned a value of 1, and rounded to the nearest integer. The summary risk score for an individual was obtained by summing the weighted scores of each of the risk factors. The overall predictive performance was measured by AUC of the ROC curve in both training and validation cohorts, with 0.5 and 1.0 indicating no and perfect predictive ability, respectively. P < 0.05 was considered statistically significant.

## Results

### Baseline characteristics of patients in training cohort

The clinical and tumor characteristics of patients in training cohort are listed in Supplemental Table 1. Briefly, the cohort consisted of 14 CK19+/GPC3+ cases, 54 CK19−/GPC3+ cases, and 49 CK19−/GPC3− cases, accounting for 12.0%, 46.2%, and 41.9% of the training data set, respectively. The microvascular invasion was present in 54 patients (46.2%), among whom 5 (4.3%) were found with the invasion of macroscopic tumor thrombi after OLT. Within three years after OLT, the total recurrence rate of patients in this cohort was 18.0%. The median time to recurrence was 12 months (range, 3–36 months). The median RFS time was 35 months (range, 1–120 months). Five (4.3%) patients from this cohort died during the follow-up without metastasis or recurrence.

### Cut-off value of serum AFP level

**Figure 2:**
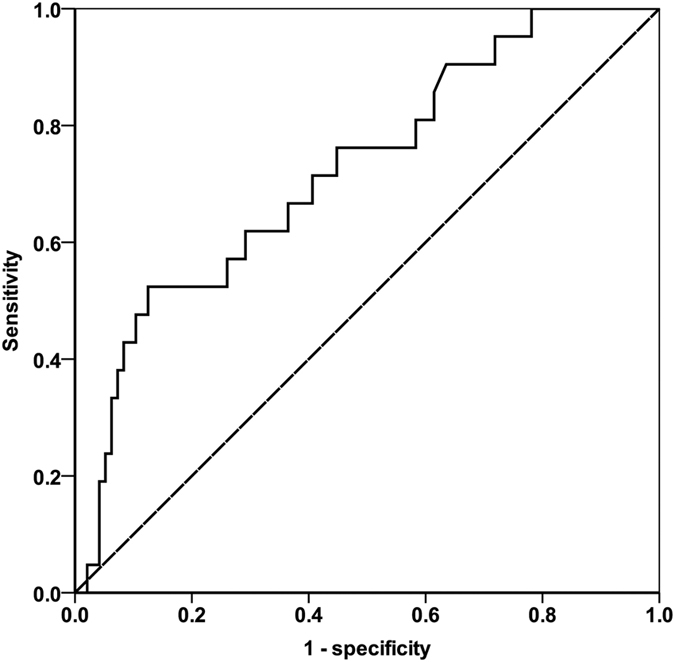
Receiver-operating characteristic curve showing sensitivity and specificity for serum alpha fetal protein cut-off values.

The pre-transplant serum AFP cut-off values in LT candidates and its impact on post-transplantation results are yet controversial. In the training cohort, 43 patients showed a serum AFP level ≤10 ng/mL, which accounted for 36.8% of all the patients. The median AFP value was 20 ng/mL (range, 1–44335). When the cut-off was determined for the point on the ROC curve that maximizes the sensitivity (52.4%) and specificity (87.5%) by Youden’s index, the optimal AFP cut-off value for tumor recurrence was 321 ng/mL. The AUC for AFP was 0.724 (95% CI = 0.604–0.843; P = 0.001) (Fig. 2).

### Univariate analyses of RFS in HCC patients of the training cohort

The RFS was compared for nine putative prognostic factors, including age, gender, presence of cirrhosis, nodule numbers, macroscopic tumor thrombi, microvascular invasion, histological grading, CK19/GPC3 expression pattern, and AFP-CV. Using the Kaplan–Meier method, univariate analysis showed that CK19/GPC3 expression pattern (overall: P < 0.001; within groups, P < 0.05), histological grading (overall: P = 0.001; within groups: well vs. poorly: 0.01 < P < 0.05, well vs. moderately: P > 0.05, moderately vs. poorly: P < 0.01), microvascular invasion(P < 0.01), and AFP-CV (P < 0.01) were significantly associated with the RFS of patients. Likewise, to the RFS of patients with well and moderately differentiated HCC groups, the similar parameters within the prognostic factors were combined into the same group. The modified bi-classification of histological grading showed high statistical significance in RFS (well + moderately vs. poorly: P < 0.01) (Table 1).

### Multivariate Cox regression analyses

Before performing the multivariate analyses, the predictor variables were assessed for multicollinearity. The VIF and tolerance did not indicate any challenging multicollinearity among the main factors (Supplemental Table 2). Therefore, four variables including the CK19/GPC3 expression pattern, histological grading (bi-classification), microvascular invasion, and AFP-CV that were significant in the univariate analysis were selected for the multivariate analysis. As shown in Table 2, the CK19/GPC3 expression pattern [CK19−/GPC3+: hazard ratio (HR) = 4.830, 95% confidence interval (CI) = 1.074–22.717, P = 0.040; CK19+/GPC3+: HR = 11.272, 95% CI = 2.291–55.464, P = 0.003] and AFP-CV > 321 ng/mL (HR = 3.224, 95% CI = 1.381–7.527, P = 0.007) were shown to be independent predictors of RFS.

### Development of recurrence prediction model

**Figure 3:**
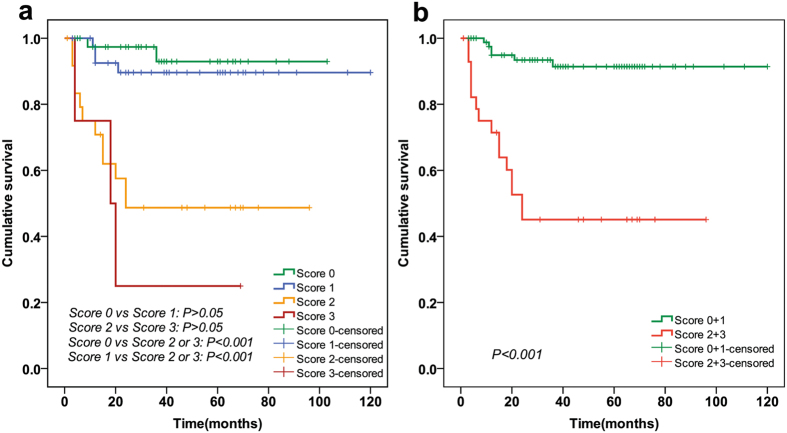
The risk factor index-based survival analysis of patients in the training cohort. According to the CK19 and GPC3 expression pattern and AFP-CV, the risk factor index was calculated and all the cases were divided into four groups (score: 0, 1, 2, and 3). (**a**) Showed that the survival curve of patients in score 0 group distinctly crossed with that of patients in score 1 group (P = 0.462). In addition, the survival curve of patients in score 2 group was very close to that of patients in score 3 group (P = 0.459). Then, the patients were further subdivided into two groups: group A (score 0 and 1) and group B (score 2 and 3). In (**b**), the log-rank test showed a significant difference between the survival curves of the two groups (P < 0.001).

According to the multivariate Cox regression result, two independent predictors were entered in risk factor scoring model to conjecture the tumor recurrence. A score for each significant predictive factor was assigned based on its coefficient value: HCC with CK19+/GPC3+, CK19−/GPC3+, and CK19−/GPC3− expression was assigned 2, 1, and 0 points, respectively. HCC with serum AFP level under the cut-off value of 321 ng/mL was assigned 0 points, and 1 was assigned for the remaining. Then, the risk factor index was calculated, and all the cases were divided into four groups (score: 0, 1, 2, and 3). As shown in Fig. 3a, the survival curve of patients in score 3 group obviously crossed with that of patients in score 2 group (P = 0.459). In addition, the survival curve of patients in score 1 group was very close to that of the patients in score 0 group (P = 0.462). Therefore, the patients were further subdivided into two groups: group A (score 0 and 1) and group B (score 2 and 3). As shown in Fig. 3b, the log-rank test demonstrated a significant difference between the survival curves of the two groups (P < 0.001). At 36 months after LT, the recurrence rate of patients with group B had reached 51.7%, which was markedly higher than that of group A (6.8%) and the average recurrence rate of MC^1^,^2^,^3^ (Table 3).

### Comparing the performance of the recurrence prediction model in the train cohort

**Figure 4:**
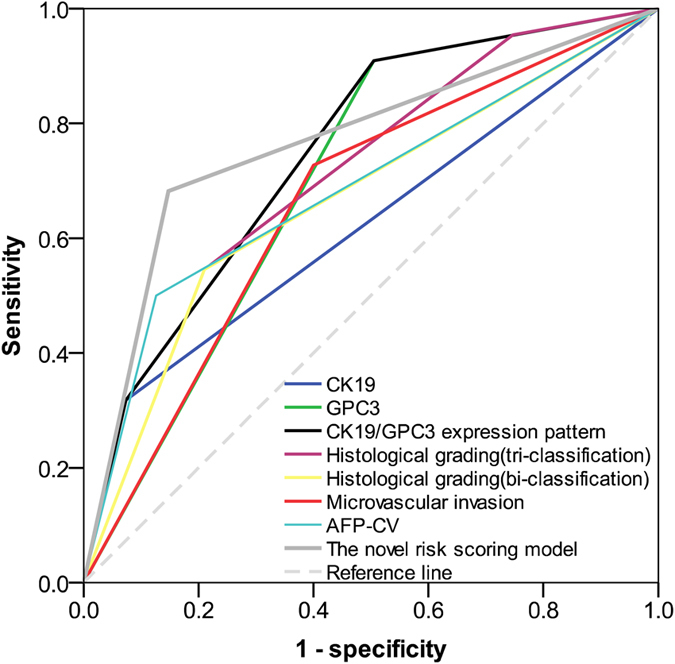
Overall predictive performance was measured by AUC of the receiver-operating characteristic curve. The AUC value of the novel risk score model was the highest comparing to that of the other elements in the training cohort.

The AUC value of CK19 was 0.622 (95% CI = 0.485–0.765; P > 0.05), that of GPC3 was 0.702 (95% CI = 0.595–0.809; P = 0.003) while that of CK19/GPC3 expression pattern was 0.749 (95% CI = 0.641–0.856; P < 0.001). The histological grading (tri-classification) was 0.707 (95% CI = 0.590–0.824; P = 0.003) and for bi-classification was 0.667 (95% CI = 0.534–0.801; P = 0.015). The microvascular invasion was 0.664 (95% CI = 0.540–0.787; P = 0.017). The AFP-CV was 0.687 (95% CI = 0.550–0.823; P = 0.006) and the novel risk score model was 0.767 (95% CI = 0.645–0.890; P < 0.001). As shown in Fig. 4, the AUC value of the novel risk score model was the highest among all the elements.

### Confirmation of prognostic ability in an independent validation cohort

**Figure 5:**
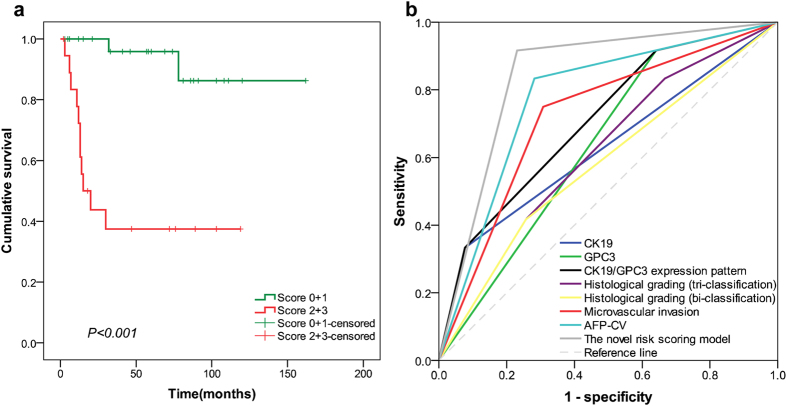
The prognostic ability of the recurrence prediction model was validated in an independent validation cohort. In (**a**), the log-rank test demonstrated a significant difference between the survival curves of the two groups (P < 0.001). (**b**) Showed that AUC value of the novel risk score model was the highest comparing to that of the other elements in the validation cohort.

The characteristics of the validation cohort were summarized at Supplemental Table 3. The median AFP was 243.6 ng/mL, which was higher than in the training cohort. The median RFS time was 41 months (range, 2–162 months). With the median recurrence time of 13 months (range, 3–32 months), the recurrence rate in this cohort was 23.5%. Four (7.8%) patients from this cohort died during the follow-up without metastasis or recurrence. As shown in Fig. 5a, the log-rank test demonstrated a significant difference between the survival curves of the two groups (P < 0.001). At 36 months after LT, the recurrence rate of patients with group B was 55.0%, which was markedly higher than that of group A (3.2%) (Supplemental Table 4). The novel model showed high performance with a good discrimination ability in the validation cohort. At 36 months after LT, the AUC value of the novel risk score model was 0.843 (95% CI = 0.720−0.966), which was the highest among all the elements and surpassed that of the training cohort (Fig. 5b).

## Discussion

In the present study, 117 HCC patients who conformed to MC and underwent OLT were investigated. Based on the combined detection of CK19 and GPC3, patients with HCC were sub-classified into CK19+/GPC3+, CK19−/GPC3+, and CK19−/GPC3−, three groups. A significant difference was found in the RFS rate after OLT between any of the three immune-phenotype groups. In addition, the AUC value of the classification of HCC by CK19 and GPC3 combined detection was higher than that of CK19 or GPC3. These characteristic suggested that HCC subtyping based on the CK19 and GPC3 expression pattern can be a more valuable indicator in predicting the recurrence of HCC patients who fulfilled the MC after OLT. The regression analysis showed that the two risk factors, including CK19/GPC3 expression pattern and AFP cutoff of ≥321 ng/mL, were the independent predictors of tumor recurrence. A simple scoring model was devised on the basis of multivariable Cox regression analytical results, which sub-classified the HCC patients into high and low-risk groups. The novel model showed high performance with a good discrimination ability in the validation cohort.

During the past two decades, the implementation of MC has considerably improved the survival in patients with HCC after LT. However, the growing experience of LT for HCC raised concerns about MC as being too restrictive and unjustified, preventing many patients from potentially curative LT. Therefore, a stepwise expansion to MC has been proposed, including the Valencia, University of California, San Francisco (UCSF), University Clinic of Navarra (CUN), and Hangzhou criteria^22^. The various groups of LT have proved that these alternative sets of criteria expanded the MC without significant loss of the post-transplant survival. However, even after fulfilling the most stringent MC, approximately 10–20% patients with HCC could develop recurrence within the first few years after LT.

Several groups have increasingly focused on exploring the biomarkers for establishing the optimal criteria fin order to predict the risk of recurrence^23^. However, the value of pre-transplant serum AFP level in the recurrence prediction is rather intriguing. A growing body of evidence has shown that the pre-transplant serum AFP is associated with the risk of tumor recurrence after LT, whereas with varied risk magnitudes depending on the defined thresholds^24^. Two independent groups reported that AFP levels >800–1000 ng/mL were associated with a high risk of recurrence after LT^25^,^26^. In the Hangzhou criteria, which expanded the MC for LT, the optimal serum AFP cut-off value for tumor recurrence was revealed to be 400 ng/mL^27^. Additionally, a recent report showed that a maximum value of AFP at a cut-off of 1000 ng/mL improved the performance of MC in predicting the tumor recurrence^28^. In the current cohort of HCC patients within MC, the ROC analysis indicated that the serum AFP level ≤321 ng/mL (AFP cut-off of >321 ng/mL was associated with approximately a 3-fold increased risk of HCC recurrence after LT) was the optimal cut-off value for HCC recurrence prediction, which was close to the value of the Hangzhou standard (400 ng/mL). However, further studies are essential to confirm the generalizability of this threshold. In addition to AFP, other biomarkers, such as serum AFP-L3 or DCP were also reported to have improved the performance of MC. Likewise, establishing the optimal thresholds of these biomarkers could potentially define a superior strategy that can be practically implemented^29^.

The correlation between the histological grading or microvascular invasion and prognosis of HCC patients has been well-reported^30^. In the present study, the univariate analysis showed that poor differentiation and microvascular invasion were significantly associated with the RFS of HCC patients after LT, which is in agreement with some previous reports^31^,^32^,^33^. However, the multivariate Cox regression analyses showed that this factor was not the independent predictor of tumor recurrence after LT. The result indicated that in comparison to the other two factors, CK19/GPC3 expression pattern, and AFP-CV can serve as powerful predictors in evaluating the recurrence of HCC patients fulfilling the MC after LT.

Combining AFP-CV and CK19/GPC3 sub-typing into MC, the novel model significantly improved the discrimination ability of individuals with high recurrence risk. Within three years after LT, comparing to the total recurrence rate of 18.0% in the training cohort and 23.5% in the validation cohort, the recurrence rate in low-risk groups of the training cohort and validation cohort were 6.8% and 3.2%, respectively. And the recurrence rate in high-risk groups of the training cohort and validation cohort were 51.7% and 55.0%, respectively. The results clearly ascribed the advantages of this model. Compared to that of any of the other elements, the highest AUC value of 0.767 in the training cohort and 0.843 in the validation cohort by the novel model exhibits its best performance in recurrence prediction.

With the frequent employment of core needle biopsy, the combining imaging technology and IHC technique, information on the size and number of tumors, as well as, the CK19/GPC3 expression can be acquired before LT, which implied that this model could be potentially used in the recipient selection. Since the subsets of patients with score 0 or 1 have lower recurrence rate as compared to that of the patients fulfilling the MC, LT might be a safer choice and should be recommended firstly if possible. For patients with score 2 or 3, when LT was selected, these subsets of patients have to encounter high early recurrence risk within the first two years after LT; strict surveillance for tumor recurrences in these patients is imperative.

The primary limitations of this study included its retrospective nature and associated issues including patient selection bias. For example, all of our patients had chronic HBV infection other than the HCV infection, which is more common in East Asian countries. Whether the model is safe and efficient in western cohorts should be clarified. In addition, for patients with infinitesimal tumors, the performance of the core needle biopsy is challenging and hence, experienced doctors are desirable. Thirdly, the survey on the recurrence of the cholangiocyte carcinoma, which presents the CK19+/GPC3− phenotype, was not included in this study. Finally, an over-prediction was observed for three year time point in both low and high risk groups probably due to higher AFP level, non-relapse death at early stage after LT and a relatively short follow-up time. A large sample size of this tumor is needed in future to establish a comprehensive model.

## Conclusion

Combing AFP-CV and CK19/GPC3 sub-typing into MC, a novel risk score model was put forward which showed a significantly improved predictive capacity for the recurrence of HCC patients after OLT. The novel model could potentially assist the select the patients before LT based on the preoperative needle core biopsy.

## Additional Information

**How to cite this article:** Feng, J. *et al*. Simple Risk Score for Prediction of Early Recurrence of Hepatocellular Carcinoma within the Milan Criteria after Orthotopic Liver Transplantation. *Sci. Rep.* **7**, 44036; doi: 10.1038/srep44036 (2017).

**Publisher's note:** Springer Nature remains neutral with regard to jurisdictional claims in published maps and institutional affiliations.

## Supplementary Material

Supplementary Tables

## Figures and Tables

**Table 1:** Univariate analysis with respect to tumor recurrence in patients of the training cohort.

Characteristic	n	Recurrence-free survival rates				P value
		6 months	12 months	24 months	36 months	
Gender						P = 0.745
Male	96	0.946 ± 0.024	0.901 ± 0.031	0.811 ± 0.043	0.795 ± 0.045	
Female	21	0.950 ± 0.049	0.835 ± 0.088	0.775 ± 0.100	0.775 ± 0.100	
Age						P = 0.955
≤50	78	0.946 ± 0.026	0.862 ± 0.041	0.802 ± 0.048	0.802 ± 0.048	
>50	39	0.949 ± 0.035	0.949 ± 0.035	0.812 ± 0.070	0.766 ± 0.080	
Cirrhosis						P = 0.574
Yes	114	0.934 ± 0.022	0.883 ± 0.029	0.805 ± 0.038	0.792 ± 0.039	
No	3	1.000	0.667 ± 0.272	0.667 ± 0.272	0.667 ± 0.272	
Macroscopic tumor thrombi						P = 0.893
Yes	5	0.953 ± 0.020	0.894 ± 0.030	0.806 ± 0.040	0.791 ± 0.042	
No	112	0.949 ± 0.020	0.895 ± 0.029	0.815 ± 0.038	0.801 ± 0.039	
Microvascular invasion						P = 0.003
Yes	54	0.962 ± 0.026	0.825 ± 0.053	0.674 ± 0.067	0.674 ± 0.067	
No	63	1.000	0.945 ± 0.031	0.926 ± 0.036	0.897 ± 0.045	
Tumor number						P = 0.224
1	92	0.943 ± 0.025	0.881 ± 0.035	0.801 ± 0.045	0.784 ± 0.047	
2	16	1.000	0.938 ± 0.061	0.938 ± 0.061	0.938 ± 0.061	
3	9	0.889 ± 0.105	0.889 ± 0.105	0.635 ± 0.169	0.635 ± 0.169	
AFP-CV						P < 0.001
≤321 ng/mL	92	0.989 ± 0.011	0.977 ± 0.016	0.889 ± 0.035	0.870 ± 0.039	
>321 ng/mL	25	0.783 ± 0.086	0.739 ± 0.092	0.508 ± 0.106	0.508 ± 0.106	
Histological grading (tri-classification)						P = 0.001
Poorly	32	0.804 ± 0.072	0.703 ± 0.083	0.625 ± 0.090	0.625 ± 0.090	
Moderately	60	1.000	0.963 ± 0.025	0.843 ± 0.051	0.816 ± 0.056	
Well	25	1.000	0.952 ± 0.046	0.952 ± 0.046	0.952 ± 0.046	
Histological grading (bi-classification)						P = 0.001
Poorly	32	0.804 ± 0.072	0.703 ± 0.083	0.625 ± 0.090	0.625 ± 0.090	
Moderately + Well	85	1.000	0.960 ± 0.023	0.874 ± 0.039	0.854 ± 0.043	
CK19 expression						P = 0.001
CK19+	14	0.846 ± 0.100	0.769 ± 0.117	0.462 ± 0.138	0.462 ± 0.138	
CK19−	103	0.950 ± 0.022	0.896 ± 0.031	0.848 ± 0.038	0.832 ± 0.040	
GPC3 expression						P = 0.001
GPC3+	68	0.895 ± 0.038	0.818 ± 0.048	0.680 ± 0.060	0.680 ± 0.060	
GPC3−	49	1.000	0.976 ± 0.024	0.976 ± 0.024	0.937 ± 0.044	
CK19/GPC3 expression pattern						P < 0.001
CK19+/GPC3+	14	0.846 ± 0.100	0.769 ± 0.117	0.462 ± 0.138	0.462 ± 0.138	
CK19−/GPC3+	54	0.906 ± 0.040	0.829 ± 0.052	0.739 ± 0.063	0.739 ± 0.063	
CK19−/GPC3−	49	1.000	0.976 ± 0.024	0.976 ± 0.024	0.937 ± 0.044	

AFP-CV: classification by AFP cut-off value; CK19: cytokeratin 19; GPC3: glypican 3.

**Table 2:** Multivariate Cox regression analysis and integer score assignment algorithm based on the β-coefficients.

Variable	β-coefficient	HR	95.0% CI (Lower-Upper)	P value	Score
CK19/GPC3 expression pattern					
CK19−/GPC3−		reference			0
CK19−/GPC3+	1.575	4.830	1.074–21.717	P = 0.040	1
CK19+/GPC3+	2.422	11.272	2.291–55.464	P = 0.003	2
AFP-CV					
≤321 ng/mL		reference			0
>321 ng/mL	1.171	3.224	1.381–7.527	P = 0.007	1

HR: hazard ratio; CI: confidence intervals; CK19: cytokeratin 19; GPC3: glypican 3; AFP-CV: classification by AFP cut-off value.

**Table 3:** Recurrence-free survival by the risk score in the training cohort.

Group	n	Estimated RFS (Mean ± Std.)				p	Recurrence risk
		6 months	12 months	24 months	36 months		
A (Score 0–1)	88	98.8% ± 0.012	93.8% ± 0.027	92.3% ± 0.030	90.3% ± 0.035	<0.001	Low
B (Score 2–3)	29	78.6% ± 0.078	71.4% ± 0.085	45.1% ± 0.096	45.1% ± 0.096		High

RFS: recurrence-free survival; Std.: Standard error.
