# Supplementary material for: Simple Risk Score for Prediction of Early Recurrence of Hepatocellular Carcinoma within the Milan Criteria after Orthotopic Liver Transplantation
Source: Sci Rep. 2017 Mar 9;7:44036. doi: 10.1038/srep44036 (PMC5343663; doi:10.1038/srep44036)
Supplement: Supplementary Tables [file srep44036-s1.doc]

**Simple Risk Score for Prediction of Early Recurrence of Hepatocellular Carcinoma within the Milan Criteria after Orthotopic Liver Transplantation**

**Jiliang Feng^1†^, Jushan Wu^2†^,** Ruidong Zhu^2^, Dezhao Feng^3^, Lu Yu^1^, Yan Zhang^1^, Dayu Bu^4^, Chenlei Li^4^, Yuyan Zhou^1^, Lianghao Si^1^, Yuhan Liu^1^, Ziwei Liang^1^, Jianing Xu^1^, Tianjun Wu^1^

**Supplemental Table 1. Baseline clinical characteristics of patients in the training cohort**

| **Variable** |  | **Value** |
| --- | --- | --- |
| **Age, years** | Median | 52.0 |
|  | Range | 29-69 |
|  | Mean±SD | 53.9±10.6 |
| **Male, n(%)** |  | 96(82.1) |
| **Cirrhosis, n(%)** |  | 114(97.4) |
| **Etiology, n(%)** |  |  |
|  | HBV infection | 106(90.6) |
|  | HCV infection | 6(5.1) |
|  | Alcohol abuse | 1(0.9) |
|  | Primary biliary cirrhosis | 1(0.9) |
|  | Autoimmune hepatitis | 1(0.9) |
|  | Budd-Chiari syndrome | 1(0.9) |
|  | Schistosome infection | 1(0.9) |
| **CK19/GPC3 expression pattern, n(%)** |  | |
|  | CK19+/GPC3+ | 14(12.0) |
|  | CK19-/GPC3+ | 54(46.2) |
|  | CK19-/GPC3- | 49(41.9) |
| **Number of tumor nodule, n(%)** |  | |
|  | 1 | 92(78.6) |
|  | 2 or 3 | 25(21.4) |
| **Histological grading, n(%)** |  | |
|  | Poorly | 32(27.4) |
|  | Moderately | 60(51.3) |
|  | Well | 25(21.4) |
| **Microvascular invasion, n(%)** | Yes | 54(46.2) |
| **Macroscopic tumor thrombi, n(%)** | Yes | 5(4.3) |
| **Histological variation, n(%)** |  | |
|  | Acinar/thin trabecular | 46(39.3) |
|  | Thick trabecular | 53(45.3) |
|  | Scirrhous | 9(7.7) |
|  | Others | 9(7.7) |

SD Error: standard error; HBV: hepatitis B virus; HCV: hepatitis C virus; CK19: cytokeratin 19; GPC3: glypican 3.

**Supplemental Table 2. Multicollinearity analyses**

| **Variable** | **Adjusted R^2^** | **Unstandardized Coefficients** | | **Standardized Coefficients** | **t** | **P** | **Tolerance** | **VIF** |
| --- | --- | --- | --- | --- | --- | --- | --- | --- |
|  |  | **B** | **Std.Error** | **β** |  |  |  |  |
| **CK19/GPC3 expression pattern** | 0.220 | 0.133 | 0.570 | 0.228 | 2.322 | 0.022 | 0.699 | 1.432 |
| **AFP-CV** |  | 0.279 | 0.084 | 0.284 | 3.339 | 0.001 | 0.931 | 1.075 |
| **Microvascular invasion** |  | 0.083 | 0.071 | 0.106 | 1.167 | 0.246 | 0.818 | 1.222 |
| **Histological grading (bi-classification)** |  | 0.086 | 0.082 | 0.098 | 1.052 | 0.295 | 0.774 | 1.293 |

Dependent Variable: recurrence; VIF:variance inflation factor; Std. Error: standard error; CK19: cytokeratin 19; GPC3: glypican 3; AFP-CV: classification by AFP cut-off value.

A tolerance of less than 0.20 and/or a VIF of 10 and above indicates a multicollinearity problem.

**Supplemental Table 3. Baseline clinical characteristics of patients** in the validation cohort

| **Variable** |  | **Value** |
| --- | --- | --- |
| **Age, years** | Median | 53.0 |
|  | Range | 35-73 |
|  | Mean±SD | 52.0±1.06 |
| **Male, n(%)** |  | 42(82.4) |
| **Cirrhosis, n(%)** |  | 46(90.2) |
| **Etiology, n(%)** |  |  |
|  | HBV infection | 42(82.4) |
|  | HCV infection | 5(9.8) |
|  | Alcohol abuse | 1(2.0) |
|  | HBV + HCV infection | 2(3.9) |
|  | Autoimmune hepatitis | 1(2.0) |
| **CK19/GPC3 expression pattern, n(%)** |  | |
|  | CK19+/GPC3+ | 7(13.7) |
|  | CK19-/GPC3+ | 29(56.9) |
|  | CK19-/GPC3- | 15(29.4) |
| **Number of tumor nodule, n(%)** |  | |
|  | 1 | 37(72.5) |
|  | 2 or 3 | 14(27.5) |
| **Histological grading, n(%)** |  | |
|  | Poorly | 15(29.4) |
|  | Moderately | 21(41.2) |
|  | Well | 15(29.4) |
| **Microvascular invasion, n(%)** | Yes | 21(41.2) |
| **Macroscopic tumor thrombi, n(%)** | Yes | 3(5.9) |
| **Histological variation, n(%)** |  | |
|  | Acinar/thin trabecular | 17(33.3) |
|  | Thick trabecular | 26(51.0) |
|  | Scirrhous | 6(11.8) |
|  | Others | 2(3.9) |

SD Error: standard error; HBV: hepatitis B virus; HCV: hepatitis C virus; CK19: cytokeratin 19; GPC3: glypican 3.

**Supplemental Table 4. Recurrence-free survival by the risk score in the validation cohort**

RFS: recurrence-free survival; Std.: Standard error

| **Group** | **n** | **Estimated RFS（**Mean±Std.**）** | | | | **p** | **Recurrence risk** |
| --- | --- | --- | --- | --- | --- | --- | --- |
|  |  | **6 months** | **12 months** | **24 months** | **36 months** |  |  |
| A(Score 0-1) | 31 | 100% | 100% | 100% | 95.7%±0.043 | <0.001 | Low |
| B(Score 2-3) | 20 | 88.9%±0.074 | 72.2%±0.106 | 43.8%±0.119 | 37.5%±0.117 |  | High |
